# Enhancing an HIV index case testing passive referral model through a behavioural skills‐building training for healthcare providers: a pre‐/post‐assessment in Mangochi District, Malawi

**DOI:** 10.1002/jia2.25292

**Published:** 2019-07-19

**Authors:** Tapiwa A Tembo, Maria H Kim, Katherine R Simon, Saeed Ahmed, Teferi Beyene, Elizabeth Wetzel, Mphatso Machika, Chrissy Chikoti, Willy Kammera, Henry Chibowa, Zinaumaleka Nkhono, Elijah Kavuta, Peter N Kazembe, Nora E Rosenberg

**Affiliations:** ^1^ Baylor College of Medicine Children's Foundation Lilongwe Malawi; ^2^ Department of Pediatrics Baylor College of Medicine Houston TX USA; ^3^ Malawi Ministry of Health Lilongwe Malawi; ^4^ University of North Carolina Project Lilongwe Malawi; ^5^ Department of Health Behavior University of North Carolina at Chapel Hill Chapel Hill NC USA

**Keywords:** HIV testing and counselling, index case finding, family referral slip, passive referral, sexual partners

## Abstract

**Introduction:**

Although knowledge of HIV positivity is a necessary step towards engagement in HIV care, more than one quarter of HIV‐positive Malawians remain unaware of their HIV status. Testing the sexual partners, guardians and children of HIV‐positive persons (index case finding or ICF) is a promising way of identifying HIV‐positive persons unaware of their HIV status. ICF can be passive where the HIV‐positive individual (index) invites a partner (or contact) for HIV testing or active where a health provider assists the index with partner notification and offers HIV testing to the partner. Strategies to improve passive ICF have not been thoroughly studied. We describe the impact of a behavioural skills‐building training to enhance healthcare workers’ (HCWs) implementation of Malawi's passive ICF programme.

**Methods:**

In June 2017, HCWs from 36 health facilities in Mangochi were oriented to Malawi's ICF programme and began implementation. In February and April 2018, a total of 573 HCWs from these facilities received further training from the Tingathe Programme. The training focused on eliciting more untested sexual contacts from indexes and better equipping indexes on issuing “family referral slips” to contacts. Monthly programmatic data were abstracted from clinical registers from October 2017 to July 2018. Monthly programmatic indicators were collected from the Index Case Testing Register and the HIV Counselling and Testing Register and were entered into a data set with one record per facility per month. T‐tests were used to compare the means of these indicators.

**Results:**

During the ten‐month study period, there were 200 facility‐months observed before and 124 facility‐months observed after training. The mean number of indexes identified per facility‐month remained stable after training (pre = 18.9, post = 21.2, *p *=* *0.74), but the mean number of sexual partners listed per facility‐month (pre = 6.3, post = 10.6, *p *<* *0.001) increased. The mean number of contacts who received HIV testing (pre = 11.1, post = 24.8, *p *<* *0.001) and the mean number of HIV‐positive contacts identified per facility‐month (pre = 1.3, post = 2.3, *p *<* *0.001) also increased.

**Conclusions:**

A brief behavioural skills‐building training impacted a range of meaningful outcomes, including identification of HIV‐positive individuals in a passive ICF programme. Such approaches could facilitate the identification of HIV‐positive persons unaware of their HIV status, a necessary step for engagement in HIV care.

## 1 Introduction

An estimated 36.9 million people were living with HIV in 2017, with substantial portions remaining unaware of their HIV status 1, 2, 3, 4. In Malawi, in spite of a generalized HIV epidemic and a mature HIV response, approximately 250,000 HIV‐positive adults remain unaware that they are living with HIV 5. This makes them unable to access antiretroviral therapy (ART) which has proven benefits for the health of the individual 6 and prevention of horizontal 7, 8, 9 and vertical transmission 10.

World Health Organization (WHO) guidelines recommend availability of HIV testing services (HTS) through a wide range of service delivery models and approaches tailored to local epidemiology and the social‐cultural context 11. Most recently, in late 2016, WHO developed guidelines recommending index‐based approaches to HTS, in which HIV‐positive persons are asked to invite their sexual contacts and family members for HIV testing 12. These index‐based approaches can be passive, in which the index is responsible for contact recruitment, or active, in which the healthcare system supports the index with contact recruitment 13. Such index‐based approaches are promoted due to their potential to improve HIV testing coverage and efficiently identify other undiagnosed HIV infections 12, 14, 15.

Malawi, in its national HIV guidelines, has adopted a passive referral model 16, in spite of local and international evidence demonstrating the enhanced effectiveness of active approaches, along with adequate safety 14, 15, 17, 18, acceptability 19, 20, 21 and cost‐effectiveness 22. Active approaches have not been pursued due to concerns of cost, complexity and social harms. It was within this context that we sought to assess whether improvements in the passive referral programme were possible through programmatic means.

We hypothesized that conducting a brief training aimed at enhancing the counselling skills of healthcare workers administering the index case testing programme in Malawi could enhance a range of programmatic outcomes. Observing indicators before and after a training, we evaluated the training's impact on the number of indexes who participated in the programme, the number of contacts they listed, the number of contacts who ultimately tested and the number of these contacts who tested HIV positive.

## 2 Methods

### 2.1 Study setting and standard of care

Mangochi district is situated in the Southeastern region of Malawi. The adult HIV prevalence among its 1.2 million inhabitants is 10.1% 23. During the study period, the Tingathe Programme (meaning “Together we can” in the local language Chichewa), a HIV programme of Baylor College of Medicine Children's Foundation Malawi, provided technical assistance to improve HIV testing services in 36 facilities in the district 24, 25, 26. Each of these 36 health facilities was operated by either the Malawi Ministry of Health, Christian Health Association of Malawi (CHAM) or private owners, and consisted of health centres and hospitals. All facilities provided free HIV testing, prevention and treatment services in line with Malawi Ministry of Health guidelines 16, 27.

During this period, healthcare workers (HCWs) at these 36 facilities were expected to implement Malawi's index case finding programme. First, HCWs were expected to ask all HIV‐positive persons or indexes (including those who were newly diagnosed and those who were already receiving anti‐retroviral treatment (ART) services at the health facilities) about the HIV status of their sexual partners, children and other household members (also known as contacts). All HTS and ART identification numbers of indexes approached were entered in a Screening Log Book to keep track of those already interviewed. They then issued one “family referral slip” for each contact with an unknown HIV status. Indexes were then expected to present these slips to their contacts and encourage them to present to the facility for HIV testing. Contacts who came to the facility were then offered HIV testing. Contacts newly diagnosed as HIV positive were linked to ART, interviewed as indexes and offered referral slips for any untested contacts.

The “family referral slip” included two sections. One section of the slip was given to the index and it contained the facility name, current date, and HIV Testing Services (HTS) serial number, and the request for the contact to present to the clinic. The second section of the slip had the same information, remained at the facility, and could be linked to the first section once the contact reported to the facility 28. This two‐part referral slip approach was used to match the contacts to the indexes upon returning to the facility through a unique identification number.

Documentation of this programme was conducted in two registers. The first was the HIV Testing and Counselling register, which captured each person who tested at the facility. Relevant to this analysis, the register contained the HIV status of the person tested and whether or not this person had presented with a family referral slip. This data source has been part of Malawi's national programme for many years. The second was the Index Case Testing register which was created in mid‐2017 by the Tingathe Programme. This listed the HTS identification number of the index and key characteristics of the contacts, including their age group and their relationship with the index. It also contained a section indicating whether the contact had returned, tested for HIV and what their HIV status was.

### 2.2 Study design and intervention training

Within these 36 facilities, the Tingathe Programme conducted a pre/post study from October 2017 to July 2018 assessing the impact of a behavioural skills‐building training aimed at improving the ability of HCWs to deliver this passive referral index case‐finding programme.

Malawi's index case‐finding programme has existed in guidelines since 2011, but had not been routinely operationalized until mid‐2017, under guidance from donors and the Ministry of Health. In June 2017, prior to the study period, Tingathe Programme staff members supported systematic implementation of Malawi's index case‐finding programme by conducting a basic orientation. The two‐hour orientation consisted of an instruction to offer family referral slips (FRS) to all persons following post‐test counselling and document it in the Index Case Testing Register. Neither was there limited education about the rationale for distributing FRS, nor was there any instruction on how to deliver these slips, demonstration on how to provide these messages, practice on delivering these messages, or feedback on the quality of the interaction. Through routine programmatic review following this basic orientation, we observed low numbers of family referral slips being distributed compared with the number of new HIV diagnoses occurring. In addition, the number of persons tested due to receiving a family referral slip was low, suggesting suboptimal implementation of index case finding. It was further determined that the basic orientation was not sufficient to equip providers to distribute FRS confidently. Thus, we decided to develop an additional training on behavioural skills‐building to enhance the ability of HCWs to implement the programme.

**Figure 1:**
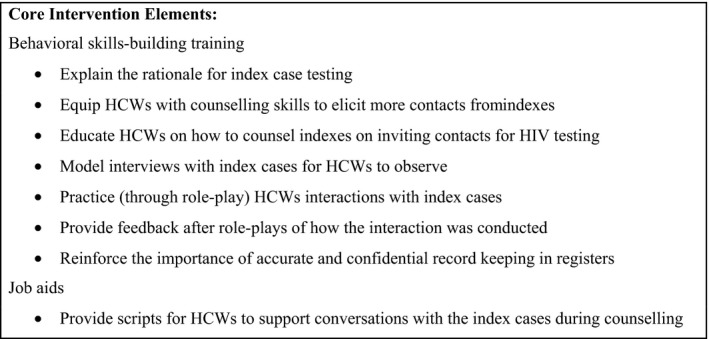
**Core elements of the intervention**

The behavioural skills‐building training was one day long and was facilitated by Tingathe programme staff members. To improve the skills of HCWs, the training had four objectives: (1) to boost understanding of the rationale for index case finding, (2) to equip HCWs with skills to effectively elicit more contacts and sensitively counsel index clients on inviting contacts for testing, (3) to model and practice HCWs interactions with indexes, and (4) to reinforce accurate and confidential record‐keeping in the registers. Specifically, through facilitator demonstrations and participant role plays, HCWs were taught to elicit motivations for indexes to offer family referral slips to contacts, support communication skills between indexes and partners, provide a range of HIV status disclosure options to indexes, and discuss how to develop action plans with indexes in support of family referral slip distribution. Furthermore, the HCWs were given a job aid with scripts to support conversations with indexes who were considering contact recruitment (Figure 1). The ultimate goal was to improve uptake of HIV testing by contacts. Our training was guided by the Information‐Motivation‐Behavioural Skills Model, which has been applied broadly to a range of HIV behaviours. Components of the training and job aid were adapted from tools created for a partner notification intervention study conducted among HIV‐positive pregnant women in Malawi 17, 29. Adaptations focused on modifying messages from a contract referral modality to a passive referral modality.

Eight of the facilities received the behavioural skills‐building training in February 2018 and the remaining 28 were trained in April 2018. There were 264 and 309 facility staff trained at the first and second trainings respectively. Training participants were recruited from a range of cadres involved in HIV service provision including Tingathe‐hired community health workers, HIV diagnostic assistants, nurses, medical assistants, clinical officers, data clerks and facility managers.

**Figure 2:**
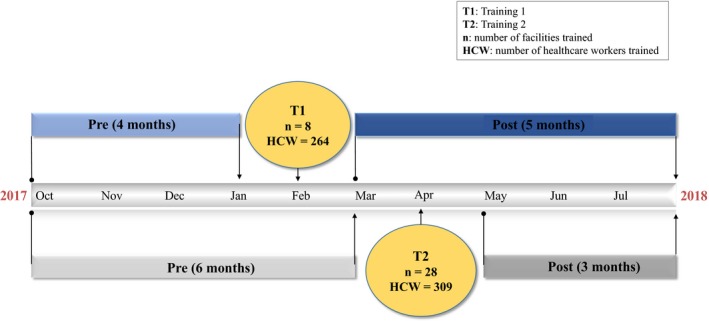
**Description of the behavioural skills‐building training timeline and number of facilities and healthcare workers trained from October 2017 to July 2018**

Each facility contributed nine months of data to the analysis. Facilities trained in February contributed four months before and five months after the training. Facilities trained in April contributed six months before and three months after the training (Figure 2). The months in which healthcare workers attended the training were excluded from analysis because it was challenging to classify them as either pre‐ or post‐intervention.

### 2.3 Data collection and management

The two primary data sources, the Index Case Testing Register and the HIV Testing and Counselling Register were abstracted monthly.

1. From the Index Case Testing registers the following variables were abstracted for this analysis:
  - Total number of index cases. These were known or newly diagnosed HIV‐positive persons who had at least one contact (defined as sexual partner, biological child or other household member) with an unknown HIV status.
  - Total number of untested contacts listed overall
  - Total number of untested contacts listed disaggregated by age: <1 year, 1 to 14 years, 15 to 24 years and > 25 years and type of relationship with index: sexual partner, biological child or guardians/other relative
  - Number of family referral slips issued
2. From the HIV Testing and Counselling register, the following monthly totals were abstracted for this analysis:
  - The number of all individuals tested at the facility,
  - The number of all individuals who tested HIV positive for the first time,
  - The number of persons (contacts) who presented for testing with a family referral slip,
    - number of contacts testing newly HIV positive (overall and by age and gender),
    - number of contacts testing HIV negative (overall and by age and gender).

These abstracted data were then entered into a database that consisted of one record per facility per month. Each record consisted of aggregate monthly data for each indicator. There was no individual‐level data. In addition to the variables described above, there was a variable for facility ID (1 to 36), facility type (public, private or CHAM), and facility level (health centre or hospital). In total, there were 10 observations for each of the 36 facilities, resulting in a dataset with 360 observations.

### 2.4 Statistical analysis

Descriptive statistics were used to summarize baseline characteristics of the health facilities. Paired t‐tests were used to compare means before and after the training. Chi‐squared tests were used to compare the proportions of all testers who presented with a family referral slip before and after the training. Unadjusted and adjusted Poisson regression models were implemented using generalized estimating equations to account for correlation by facility. Incidence rate ratios and ninety‐five percent confidence intervals comparing all programmatic indicators pre‐ and post‐training were reported. Models were adjusted for site‐related variables, including facility type and level, as well as for calendar month (1 to 10) to control for potential confounding. Data were analysed using Stata 13.

### 2.5 Ethical approval

The study was approved by the National Health Science Research Committee in Malawi and the Baylor College of Medicine Institutional Review Board under a protocol granting review of routine Tingathe programme data. Because all data were routine programmatic facility‐level data, with no individual information collected, patient‐level consent was not obtained.

## 3 Results

### 3.1 Health facility characteristics

The overall data set contained 200 total facility months before training and 124 facility months after training. Thirty‐six months were omitted since this is when training occurred. Twenty‐two facilities were operated by the Ministry of Health, 11 were operated by CHAM and three were private. Four were secondary level hospitals and 32 were health centres (Table 1). Pre‐ and post‐changes were similar across the two training groups.

**Table 1:** Facility characteristics

Variable	n (%)
Training group	
Training 1 in February 2018	8 (22)
Training 2 in April 2018	28 (78)
Months pre‐ or post‐training	
Pre‐training	200 (62)
Post‐training	124 (38)
Facility type	
Ministry of Health	22 (61)
Christian Health Association of Malawi (CHAM)	11 (31)
Private	3 (8)
Facility level	
Health Centre	32 (89)
Hospital	4 (11)

**Figure 3:**
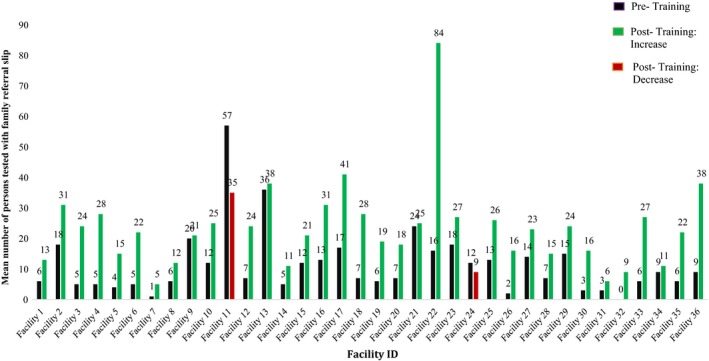
**Mean number of contacts who returned with a family referral slip per facility per month pre‐ and post‐training**

**Figure 4:**
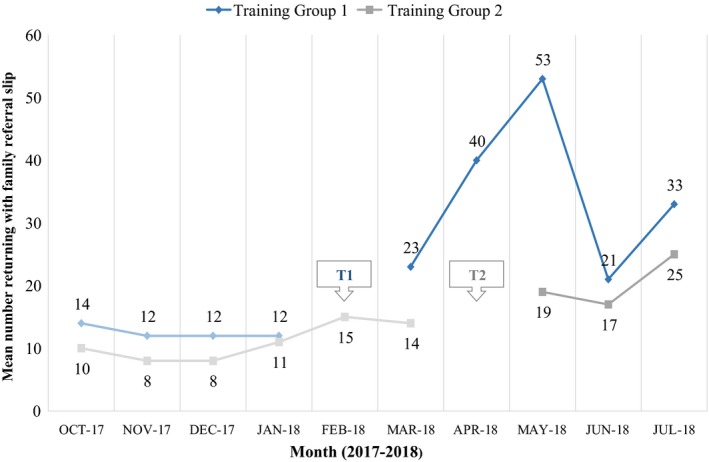
**Mean number of contacts who returned with family referral slip by training group**

Following the training, the mean number of persons returning with a family referral slip increased from 11 persons pre‐training to 25 persons post‐training (*p *<* *0.001). This finding was robust: an increase was observed in 34 of the 36 facilities (Figure 3). Similarly, the overall monthly number of persons returning with a family referral slip was higher in every month post‐training relative to pre‐training (Figure 4). This upward trend was observed for children and adults.

### 3.2 Comparison of key indicators pre‐ and post‐training

Based on the Index Case Testing register, we observed no increase in the mean number of index cases per facility per month before and after training (pre = 18.9, post = 21.2, *p *=* *0.74). We observed no increase in the mean number of contacts listed per facility per month before and after the training (pre = 35.9, post = 42.2., *p *=* *0.41), with considerable variability by age group and relationship with the index. There was no increase in the mean number of contacts by age <1 year old (pre = 0.6, post = 0.3, *p *=* *0.06), 1 to 14 years old (pre = 24.1, post = 26.7, *p *=* *0.69) or 15 to 24 years old (pre = 4.5, post = 5.1, *p *=* *0.62). However, there was a notable increase in mean number of contacts listed who were ≥ 25 years old (pre = 5.8, post = 10.2, *p *<* *0.001). Similarly, there was no increase in the mean number of biological children (pre = 28.1, post = 30.1, *p *=* *0.85) or guardians/other (pre = 1.3, post = 1.4, *p *=* *0.99), but there was a substantial increase among the mean number of untested sexual partners (pre = 6.3, post = 10.6, *p *<* *0.001) (Table 2). Mean number of contacts listed by index was higher in hospitals compared with health centres (*p *=* *0.001).

**Table 2:** A comparison of means per clinic per month pre‐ and post‐training

Source	Mean pre	Mean post	*p*‐value	Unadjusted poisson regression	Adjusted poisson regression^a^
Index case testing register					
Index cases per month	18.9	21.2	0.74	0.99 (0.81 to 1.20)	1.29 (0.76 to 2.17)
Contacts listed by index	35.9	42.2	0.41	1.07 (0.89 to 1.28)	1.49 (1.00 to 2.21)
<1 year	0.6	0.3	0.06	0.46 (0.22 to 0.97)	0.30 (0.05 to 1.75)
1 to 14 years	24.1	26.7	0.69	1.04 (0.85 to 1.28)	1.55 (0.99 to 2.42)
15 to 24 years	4.5	5.1	0.62	0.97 (0.71 to 1.32)	1.43 (0.86 to 2.37)
≥25 years	5.8	10.2	<0.001	1.44 (1.24 to 1.68)	1.55 (1.09 to 2.20)
Sexual partners	6.3	10.6	<0.001	1.39 (1.20 to 1.60)	1.54 (1.08 to 2.17)
Biological children	28.1	30.1	0.85	0.99 (0.81 to 1.22)	1.49 (0.95 to 2.33)
Guardians/other	1.3	1.4	0.99	1.01 (0.65 to 1.59)	1.39 (0.48 to 3.99)
Family referral slips issued to indexes	35.7	42.2	0.41	1.07 (0.89 to 1.30)	1.48 (0.95 to 2.29)
HIV testing and counselling register					
Persons tested	743.9	854.9	0.38	1.03 (0.96 to 1.10)	0.79 (0.67 to 0.93)
Persons newly tested HIV positive	24.7	30.3	0.70	1.02 (0.96 to 1.07)	0.92 (0.79 to 1.07)
Persons tested with family referral slip	11.1	24.8	<0.001	2.12 (1.59 to 2.83)	1.39 (0.93 to 2.08)
Male	5.7	13.6	<0.001	2.23 (1.61 to 3.09)	1.55 (0.93 to 2.58)
Female	5.5	11.1	<0.001	1.97 (1.51 to 2.56)	1.28 (0.90 to 1.80)
<15 years	6.6	11.2	<0.001	1.67 (1.17 to 2.38)	0.88 (0.59 to 1.31)
≥15 years	4.6	13.6	<0.001	2.75 (2.10 to 3.60)	2.48 (1.27 to 4.86)
Persons tested HIV negative with a family referral slip	9.9	22.5	<0.001	2.17 (1.60 to 2.95)	1.45 (0.96 to 2.21)
Persons newly tested HIV positive with family referral slip	1.3	2.3	<0.001	1.57 (1.23 to 1.99)	1.07 (0.64 to 1.80)
Male	0.8	1.4	<0.001	1.69 (1.18 to 2.42)	1.15 (0.65 to 2.05)
Female	0.6	0.9	0.02	1.5 (1.11 to 2.03)	1.12 (0.50 to 2.53)
<15 years	0.4	0.4	0.77	1.11 (0.69 to 1.78)	0.81 (0.32 to 2.01)
≥15 years	1.0	1.9	<0.001	1.78 (1.41 to 2.26)	1.25 (0.69 to 2.31)

a Adjusted for calendar month, facility level and facility type.

With regard to indicators in the index case testing register, in unadjusted and adjusted analyses, we observed a significant increase in contacts listed that were ≥ 25 years and sexual partners. In adjusted analysis, we also observed 1.5 times the number of overall contacts listed by index in the post‐period relative to the pre‐period (1.00, 2.21). We did not observe significant increases in contacts listed that were <1, 1 to 14, 15 to 24 years, biological children and guardians/other.

Based on the HIV Testing and Counselling register, the mean number of persons tested per month was comparable before and after the training (pre = 743.9, post = 854.9, *p *=* *0.38). Mean number of persons newly identified as HIV positive did not increase (pre = 24.7, post = 30.3, *p *=* *0.70).

The mean number of contacts who tested HIV positive with a family referral slip increased from 1.3 pre‐training to 2.3 post‐training (*p *<* *0.001). This increase was greater in male contacts than female contacts and was apparent in adult contacts, but not paediatric contacts.

With regard to indicators in the HTC register, in unadjusted and adjusted analyses, we observed a significant increase in persons ≥ 15 years tested with family referral slip. In the other subgroup analyses (number of men, number of women, number of persons <15 years, number of persons testing HIV negative and number of persons testing HIV positive), we observed significant increases in unadjusted analyses, but in the adjusted regression analyses, these trends were attenuated considerably.

Two additional indicators improved following the training: the proportion of all persons tested through a family referral slip (pre = 1.5%, post‐training = 3.3%, *p *=* *0.28) and the ratio of contacts who returned to the number of family referral slips issued (pre = 1:3.3, post = 1:1.7, *p *<* *0.001). Of the total referral slips distributed, 30% pre‐ and 59% post‐training returned (*p *<* *0.001).

One additional descriptive observation is that both before and after training, the proportion of persons who were HIV positive was higher among contacts presenting with a family referral slip (pre = 11.9%, post = 9.4%) than among the general population of persons testing at these facilities (pre = 3.3%, post = 3.5%). This trend was especially pronounced among adults presenting with a family referral slip (pre = 21%, post = 14%).

## 4 Discussion

In an assessment of 36 Malawian health facilities observed before and after a one‐day behavioural skills‐building training, three meaningful programmatic indicators improved. First, HIV‐positive index patients named more potential contacts, especially adult sexual partners. Second, a higher number of adult contacts returned for HIV testing. Third, more HIV‐positive adult contacts were identified. These findings were robust across different facility types and months under observation.

In the light of recent WHO guidelines promoting index‐based approaches, this finding is timely and addresses important gaps in knowledge. Most research has focused on comparing active index‐based approaches to passive ones 14, 17, 30. To the best of our knowledge, this is the first rigorous assessment evaluating an enhanced passive referral approach in a low‐resource setting within the context of routine programme delivery. Enhanced passive approaches are appealing in such settings, as they are relatively inexpensive and typically do not require additional staffing, communication or transportation resources. Similarly, a one‐day training is neither costly nor burdensome to facilities or implementing partners, making this approach easy to replicate. Enhanced passive referral strategies may serve as a natural next step towards improving index‐based programmes in similar settings.

This analysis contributes to the first “90” in Malawi's “90‐90‐90” targets, referring to 90% of HIV‐positive individuals being aware of their status, 90% of this group being engaged in HIV care, and 90% of these persons being virally suppressed. Malawi has struggled to reach the first target, with a substantial share of HIV‐positive individuals remaining unaware of being HIV positive. This is especially true for men 5. Our training resulted not only in more contacts detected, but also in more contacts who were HIV positive, a trend that was more apparent among men. Additionally, in both the pre‐ and post‐periods, the proportion of contacts who were HIV positive exceeded the proportion of overall testers who were HIV positive, an observation that was especially apparent in adults. The efficiency of index‐based programmes at detecting HIV‐positive persons has been observed previously 18. The underlying rationale is a basic epidemiologic principle: each HIV‐positive person contracted HIV from a past or current contact and could pass it to a current or future contact. Thus, contacts of HIV‐positive persons are much more likely to have HIV‐positive contacts than persons in the general population. These results reinforce the value of index‐based approaches for finding the remaining HIV‐positive persons unaware of their HIV status. Our findings also suggest that it can be an effective strategy for closing the HIV case finding gap among men.

The greater improvement in adult indicators relative to paediatric indicators is noteworthy. This discrepancy is likely due to two factors. First, in the pre‐period indexes primarily listed paediatric contacts and these contacts accounted for the majority of those presenting for testing. Thus, there was less room for improvement among this group. Second, in the light of this observation, the training was primarily focused on eliciting more sexual contacts from indexes and supporting indexes with the invitation process. Through facilitator demonstrations and participant role plays, the training was designed to teach healthcare workers to more comfortably discuss sexual partnerships, support index patients with HIV status disclosure and help index patients discuss clinic presentation with sexual partners.

The use of routine programmatic data is both a strength and limitation in this analysis. Importantly, this data source reflects the full patient population in a large number of health facilities, resulting in very broad generalizability. There were no significant differences in the number of referral slips distributed per facility per month between government and private facilities, hospitals and health centres or those in training groups 1 and 2. Our intervention improved the mean number of contact who returned in 34 of the 36 facilities. The difference in the two facilities does not appear to be a function of training group, facility level or facility type. However, our design makes other questions of interest difficult to address. For example, due to the absence of individual‐level data, it is not possible to understand which index‐level factors were associated with contact elicitation and which index and contact characteristics were associated with contact presentation. Therefore, future research is required to address this. We could observe the magnitude of the training's impact, but have a limited understanding of how it worked. We do not know which part(s) of the training were most effective, warranting further investigation.

As expected, some indexes who received referral slips were probably not ready to disclose their HIV status to contact(s) due to fear of abandonment, violence or stigma. This may have resulted in some referral slips not being distributed and thus contacts not reporting for HTS. It is also possible that some contacts reported with no slips or accessed testing at a health facility outside of our sampled 36 facilities. Contacts were encouraged to bring the referral slip with them when they came to the facility for an HIV test in order to link them to the index. For contacts presenting for HTS without a referral slip, it was difficult to determine that they were testing as part of the ICF programme and not as part of other HIV testing services offered at the facility. Thus, we may have undercounted the number of contacts tested as part of the ICF programme. In addition, we were only able to measure contacts who presented with a referral slip at the facilities and had the FRS box ticked in the register. Therefore, additional efforts are required to increase the ascertainment of contacts who presented, but may not have been captured.

One potential concern is that the training improved the documentation of the programme, rather than the programme itself. However, this is unlikely to be the primary explanation for these findings. The majority of health facility staff who attended the behavioural skills‐building training had received the basic orientation already and overall documentation was good. This was evidenced by certain indicators (e.g. number of index cases) remaining stable, while only those indicators that were the target of the training improved. A second potential concern is that the observed increases were due to secular changes over time, rather than due to the training itself. The attenuated findings in some of the adjusted analyses, which control for time, suggests this may be a partial explanation. A related concern is that modelled estimates are imprecise, and thus not all modelled estimates were statistically significant at an alpha level of 0.05. This is expected given the short duration of follow‐up, the rare nature of HIV‐positive outcomes, and the number of covariates included in the sample. As such, to assess the optimal impact of this intervention, replication of this training in more facilities over longer periods would enhance the strength of these inferences. Nonetheless, the positive results observed in each month and in nearly every facility support the interpretation that the training had an impact.

## 5 Conclusions

In summary, our analysis shows that it is possible to impact a number of meaningful programmatic indicators across a broad range of facilities with a low‐cost one‐day training. Such findings offer a promising step towards enhancing index case finding in low‐resource settings.

## Competing interests

The authors declare that they have no competing interests.

## Authors’ contributions

TAT, MHK, NER and KS designed the study, interpreted the data and wrote the manuscript. TAT analysed the data under guidance of NER. SA and PNK critically reviewed the study design and the manuscript. TB, EW, MM, CC, WK, HC, ZN and EK reviewed the manuscript. All authors contributed to the writing of the manuscript and approved the final manuscript for publication.
